# mirTarCLASH: a comprehensive miRNA target database based on chimeric read-based experiments

**DOI:** 10.1093/database/baaf023

**Published:** 2025-04-05

**Authors:** Tzu-Hsien Yang, Xiang-Wei Li, Yuan-Han Lee, Shang-Yi Lu, Wei-Sheng Wu, Heng-Chi Lee

**Affiliations:** Department of Biomedical Engineering, National Cheng Kung University, No.1, University Road, Tainan 701, Taiwan; Medical Device Innovation Center, National Cheng Kung University, No.1, University Road, Tainan 701, Taiwan; Department of Electrical Engineering, National Cheng Kung University, No.1, University Road, Tainan 701, Taiwan; Department of Electrical Engineering, National Cheng Kung University, No.1, University Road, Tainan 701, Taiwan; Department of Electrical Engineering, National Cheng Kung University, No.1, University Road, Tainan 701, Taiwan; Department of Electrical Engineering, National Cheng Kung University, No.1, University Road, Tainan 701, Taiwan; Department of Molecular Genetics and Cell Biology, University of Chicago, 920 E. 58th Street, Suite 1106, Chicago, IL 60637, USA

## Abstract

MicroRNAs (miRNAs) can target messenger RNAs to control their degradation or translation repression effects. Therefore, identifying the target and binding sites of different miRNAs is essential for understanding miRNA functions. To investigate these interactions, researchers have employed the cross-linking, ligation, and sequencing of hybrids (CLASH-seq) and similar CLASH-like approaches to generate chimeric reads formed by miRNAs and their targeting segments. These chimeric reads allow for the direct extraction of both the miRNA–target gene pairs and their corresponding binding sites. Nevertheless, these studies lack user-friendly platforms for researchers to investigate these interactions efficiently, thus hindering scientists’ ability to explore miRNA functions. To address this gap, we developed mirTarCLASH, a comprehensive database that deposits 502 061/322 707/224 452 unique hybrid reads from human/mouse/worm miRNA chimeric read-based experiments. In mirTarCLASH, the chimera analysis algorithm ChiRA and two distinct binding site inference tools, RNAup and miRanda, were adopted to facilitate the exploration of miRNA–target pairs derived from CLASH-like experiments. Compared with existing similar repositories, mirTarCLASH further enables several confidence evaluation filters with visualization functions for the extracted results. The results can be further refined based on the key properties of the miRNA targeting sites, including read depths, numbers of supporting algorithms, and cross-linking-induced mutations, to enhance confidence levels. In addition, these miRNA-binding sites are visually represented through an integrated transcript atlas. Finally, we demonstrated the biological applicability of mirTarCLASH via the well-characterized example interaction between *cel-let-7-5p* and *lin-41* in *Caenorhabditis elegans*, showcasing the potential of mirTarCLASH to provide novel insights for subsequent experimental research designs. The constructed mirTarCLASH database is freely available at https://cosbi.ee.ncku.edu.tw/MirTarClash.

**Database URL**: https://cosbi.ee.ncku.edu.tw/MirTarClash

## Introduction

MicroRNAs (miRNAs) are RNA sequences of 18–26 nucleotides that participate in diverse physiological and pathological processes [1]. At the molecular level, mature miRNA sequences can form RNA-induced silencing complexes with Argonaute (AGO) to target transcripts, triggering messenger RNA (mRNA) post-transcriptional degradation or translation repression [2]. Aberrant miRNA targeting can have detrimental effects on cells. For example, the altered expression of several miRNAs can lead to the oncogenesis of several known cancers [3]. Considering their vital roles in cells and even human diseases, miRNAs are currently the key focus of gene expression research and hold promising potential as disease biomarkers.

Despite the critical roles of miRNA targeting, identifying the target sites of miRNAs remains challenging. In early studies, biologists investigated the canonical miRNA-binding rule based on perfect complementary base pairing within the seed regions (positions 2–7/8) of miRNAs [4]. However, subsequent research revealed several exceptions to this rule, leading to the discovery of noncanonical binding mechanisms. These noncanonical rules include the imperfect seed pairing complemented by the pairing of perfect center sites (positions 4–15) [5] or 3ʹ compensatory regions [6]. Beyond binding rules, canonical miRNAs were initially thought to primarily target 3ʹ untranslated regions (3ʹUTRs). However, recent evidence pointed out that certain miRNAs can also interact with the 5ʹ end of the target transcripts [7]. Moreover, cross-species diversities have also been observed for miRNA targeting [8]. Given the complexity of miRNA-binding mechanisms, mapping miRNA targets across different species is essential for advancing our knowledge of post-transcriptional regulation.

Numerous experimental protocols have been developed to identify the target sites of miRNAs at the transcript level. Traditional methods, such as luciferase fusion reporter assay combined with quantitative real-time polymerase chain reaction and Western blotting, can confirm miRNA–target interactions and distinguish between mRNA degradation and translational inhibition [9]. However, these approaches are limited to analyzing few miRNA–target interactions. To enable high-throughput miRNA target investigation, researchers have developed several chemical cross-linking-based protocols followed by sequencing, allowing transcriptome-wide screening of miRNA-binding events [10]. Among these, AGO-CLIP (cross-linking and immunoprecipitation) can isolate the miRNA-bound transcript segments to generate the genome-wide miRNA–target maps [11]. While CLIP-based sequencing can effectively capture the *in vivo* miRNA-binding events, predicting the exact miRNA involved in each targeting interaction is still required [12]. Later, an additional ligation step was introduced, enabling the identification of miRNA–target chimeras [13]. Examples of chimera-based techniques include CLASH (cross-linking ligation and sequencing of hybrids) [14] and CLEAR-CLIP (covalent ligation of endogenous Argonaute-bound RNAs, cross-linking and immunoprecipitation) [15] methods. These methods, through rigorous data processing steps, allow for the precise identification of miRNA–target segment pairs from the chimeric read results.

Several human resources have been devoted to collecting known miRNA–target pairs. DIANA-TarBase [16] and miRTarBase [17] are the top two most comprehensive databases for constructing miRNA–target maps. Besides manual curation of the existing small-scale experiments, recent updates to both databases have incorporated results of CLIP-seq and CLASH-seq experiments. In miRTarBase, only CLIP-seq results were gathered. Due to the nature of CLIP-seq experiments, the regulatory miRNAs were inferred rather than directly identified for these binding events, resulting in predicted miRNA–target interactions. In contrast, DIANA-TarBase has recently expanded its content to include both CLIP-seq and CLASH-seq results. Despite these expansions, the proposed analysis pipelines for CLASH datasets remain rudimentary. Furthermore, as studies reported that noncanonical miRNA targeting sites identified from CLASH may be functionally insignificant, additional analysis filtering is required to assess the confidence of the chimeric read-derived interactions. Given these unsolved challenges, a comprehensive database that deposits miRNA–target pairs from published CLASH experiments with available confidence evaluation filters would greatly contribute to the study of miRNA targeting mechanisms.

In this study, we developed mirTarCLASH, a database that integrates CLASH-like experiments from humans, mice, and worms to provide a comprehensive atlas of miRNA regulation. In mirTarCLASH, miRNA–target pairs and their binding sites are systematically identified through ChiRA. Furthermore, mirTarCLASH offers detailed annotations of the identified targeting sites, including targeting site locations, read depths, the number of supporting algorithms, estimated binding energies, and cross-linking-induced mutation sites (CIMSs) caused by reverse transcriptase bypasses and read-through [18]. These annotations serve as confidence indicators for each chimeric read result. In particular, CIMSs confirm the existence of cross-linking in chimeric reads, thereby increasing the reliability of the identified miRNA target sites. To facilitate the investigation of the miRNA-binding events probed in CLASH-like experiments, the database provides a user-friendly platform for searching miRNA-binding sites by gene or miRNA of interest, with options to filter the results based on specific miRNA targeting properties. Finally, we showcased the biological applicability of mirTarCLASH to miRNA studies through the well-characterized *cel-let-7-5p*-binding sites on *lin-41*, which include sites with both canonical and noncanonical interactions. The constructed database mirTarCLASH can be accessed at https://cosbi.ee.ncku.edu.tw/MirTarClash.

## Materials and Methods

### Overview of mirTarCLASH

**Figure 1.**
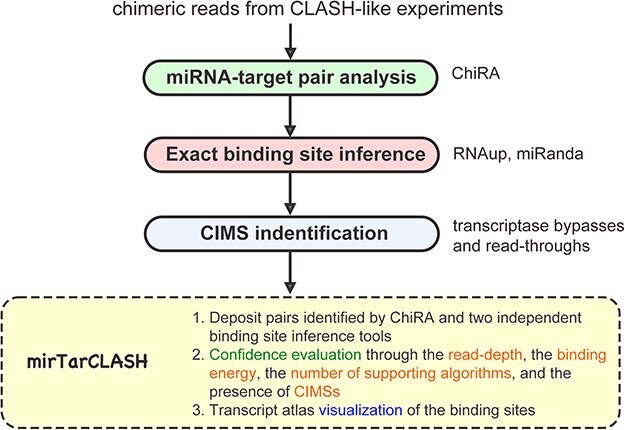
The overview of mirTarCLASH.

mirTarCLASH is a database that deposits comprehensive chimeric read-based miRNA–target pairs along with corresponding confidence metrics. The overview of mirTarCLASH is shown in Fig. 1. In its current version, mirTarCLASH integrates the CLASH-like experimental datasets for humans, mice, and worms. For each experiment, the well-established chimera analysis pipeline ChiRA was used to extract the miRNAs and their target mRNA sequences from chimeric reads. Then, the precise binding sites for each miRNA–target sequence pair were computed by two RNA–RNA interaction identification algorithms, miRanda and RNAup. To aid users in evaluating the confidence of each deposited miRNA–target sequence pair, mirTarCLASH provides detailed annotations of the chimeric reads, including the binding energy, the read depth, the number of supporting algorithms, and the presence of CIMSs. These confidence metrics enhance the reliability of the identified miRNA–target sequence pairs. The data collection and processing steps are further detailed in the following subsections.

### Data collection

In the current version of mirTarCLASH, we collected CLASH-like experiments conducted in *Homo sapiens, Mus musculus*, and *Caenorhabditis elegans*. The miRNA sequence information for all three species was retrieved from miRbase [19] (Release 22.1, downloaded on 8 January 2023). Protein-coding transcripts for *H. sapiens* (GRCh38.p13) and *M. musculus* (GRCm39) were adopted from the GENCODE database [20]. For *C. elegans*, the transcript annotations were downloaded from WormBase (version WS286) [21]. In all three species, ncRNA targets were not considered in the subsequent steps. To identify the miRNA–target sequence pairs from chimeric read-based experiments, .fastq files for each experiment were downloaded from the Sequence Read Archive (SRA) database. For humans, we gathered the CLASH experiments performed by Helwak *et al*. [14] in HEK293 cells and the CLEAR-CLIP experiments conducted by Moore *et al*. [15] in hepatoma cells. Both CLASH and CLEAR-CLIP protocols incorporate the ligation step that facilitates the detection of miRNA–target sequence chimeric reads. Moore *et al*. also applied the designed CLEAR-CLIP protocol to the mouse brain cells. Hence, we also downloaded the experimental .fastq files for the mouse brain CLEAR-CLIP results. Finally, for *C. elegans*, the AGO individual-nucleotide resolution cross-linking and immunoprecipitation (iCLIP) experimental results were adopted in mirTarCLASH. Although there is no ligation step in the iCLIP protocol, it was shown that the iCLIP experiments can also fortuitously produce miRNA–target chimeric reads [22]. Based on this observation, we gathered the iCLIP chimeric reads from the work of Broughton *et al*. [22]. The SRA run IDs of these CLASH-like experiments are summarized in Table 1.

**Table 1.** Catalogs of SRA run IDs for the chimeric read-based experiments

Species	No. of unique hybrid reads	Experimental Types	SRA ID
Human	502 061	CLASH-seq	SRR959751, SRR959752, SRR959753, SRR959754, SRR959755, SRR959756, SRR959757, SRR959758, SRR959759
		CLEAR-seq	SRR2413156, SRR2413157, SRR2413158, SRR2413159, SRR2413160, SRR2413161, SRR2413162, SRR2413163, SRR2413164, SRR2413165, SRR2413166, SRR2413167, SRR2413168, SRR2413169, SRR2413170, SRR2413171, SRR2413172, SRR2413173, SRR2413174, SRR2413175, SRR2413176, SRR2413177, SRR2413178, SRR2413179, SRR2413180, SRR2413181, SRR2413182, SRR2413183, SRR2413184, SRR2413185, SRR2413186, SRR2559259, SRR2559274
Mouse	322 707	CLEAR-seq	SRR2413277, SRR2413278, SRR2413279, SRR2413280, SRR2413281, SRR2413282, SRR2413283, SRR2413284, SRR2413285, SRR2413286, SRR2413287, SRR2413288, SRR2413289, SRR2413290, SRR2413291, SRR2413292, SRR2413293, SRR2413294, SRR2413295, SRR2413296, SRR2413297, SRR2413298, SRR2413299, SRR2413300, SRR2413301, SRR2413302
Worm	224 452	iCLIP-seq	SRR3882724, SRR3882728, SRR3882949, SRR3882950, SRR3882951

### Data processing of the chimeric reads to extract miRNA–target pairs and binding sites

For each .fastq file containing chimeric reads, we first remove the artificially added adapter and barcode sequences and filter out reads with low-quality scores using Cutadapt [23]. We then calculate the read count for each chimeric read. Since different chimera analysis tools employ varying heuristics in the calculation processes, we utilized the wildly used chimera analysis tool ChiRA [24] in mirTarCLASH to comprehensively and systematically analyze the CLASH-like experimental data and identify miRNA–target segment pairs. ChiRA adopts BWA [25] as the aligner for read mapping and merges overlapping read loci to improve the handling of multi-mapped reads. For each chimeric read, ChiRA extracts two to three potential miRNA–target pairs. Default parameters were applied when processing the CLASH-like datasets with ChiRA.

After identifying the miRNAs and their corresponding target sequence segments from the chimeric reads, mirTarCLASH further inferred the potential binding sites of each miRNA on its target sequence segment. Since CLASH and CLIP protocols include an RNase treatment step to fragment the RNAs, parts of the miRNA-binding sequences may be lost due to RNA degradation. To recover these potentially lost binding sequences, we extended the identified miRNA target segment both upstream and downstream on their residing transcript by the length of the miRNA, ensuring that the complete binding sites were included in the sequence segment. In order to pinpoint the exact binding sites, mirTarCLASH employed two RNA–RNA interaction prediction tools (RNAup [26] and miRanda [27]) that adopt different binding rule heuristics. RNAup considers the target sites of a given miRNA by computing the minimum free co-folding energy between the miRNA and the extended sequence segment extracted from a chimeric read. In miRanda, it first locally aligns a given miRNA to the extended sequence segment and scores these alignments. Then, the thermodynamic stability of the miRNA and sequence duplexes based on these alignments is evaluated. mirTarCLASH chose the best-aligned results, which usually favor canonical binding events, from miRanda. mirTarCLASH deposited all the binding sites identified by RNAup and miRanda.

### Filters to help reinforce the confidence of the identified miRNA-binding events

In the last step, mirTarCLASH provides filters to help users evaluate the confidence of the identified miRNA-binding sites. These filters consider read depths, binding energies, and CIMSs. Generally, higher read depths increase confidence in a given miRNA–target pair. CIMSs are the base substitutions or deletions caused by reverse transcriptase bypasses and read-throughs at cross-linking sites [18]. The reverse transcriptase-induced mismatches are believed to result from the residues of miRNA-mediated regulatory proteins, thus reinforcing the existence of the identified miRNA-segment binding sites in living cells. These read-through events can be reflected by mismatches or bulges, which correspond to substitutions and deletions, respectively, in the reference-read alignment. We used Bowtie2 [28] to remap the sequences of miRNA–target pairs identified by chimera analysis tools back to the reference genomes. Mismatches and deletions in the alignment results were recorded as CIMS indicators to enhance the confidence assessment of miRNA-binding events.

### Database implementation

mirTarCLASH was implemented using Python (version 3.5.2) with the Django framework (version 1.11.1). The core database service was constructed using the SQLite3 (version 3.11.0) database management system. Finally, the database user interface that ensures a responsive and interactive experience is powered by several JavaScript libraries (e.g. JQuery, Bootstrap, d3.js, and DataTables).

## Results and Discussions

### Database interface

**Figure 2.**
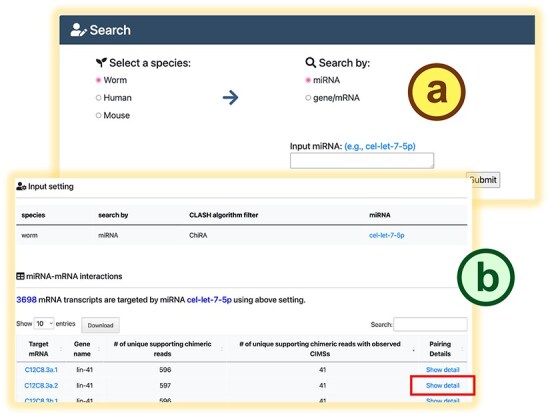
The search mode in mirTarCLASH. (a) The query settings. (b) The query result table. Click the “Show detail” link, and the detailed chimera analysis information for the pair will be provided.

mirTarCLASH is a database that compiles miRNA–target pairs and binding sites identified from CLASH-like experiments. It also features a user-friendly interface that enables visualization of miRNA-binding sites on the transcripts. Two basic use modes are offered in mirTarCLASH (Fig. 2): (i) search mode and (ii) browse mode. For each mode, detail pages (the search detail page and the browse detail page) are available for in-depth analysis of miRNA–target interactions.

**Figure 3.**
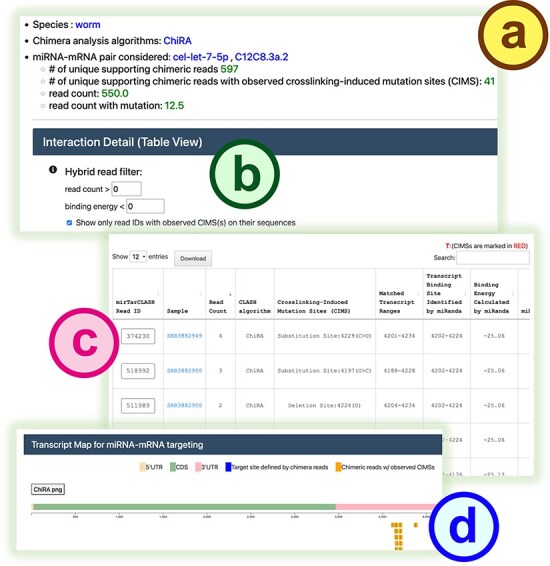
The detail page in mirTarCLASH. (a) The query settings for the miRNA–mRNA pair under investigation. (b) The confidence filters for the listed pairs. The detailed search results of a specific miRNA–target transcript pair in the tabular (c) and graphical (d) view.

In the search mode (Fig. 2a), users can provide an miRNA of interest to retrieve its target mRNAs and binding sites or input a gene/transcript of interest to obtain the miRNAs probed to bind it in CLASH-like experiments. After submitting a query, mirTarCLASH generates a summary table (Fig. 2b) listing all deposited miRNA–target gene pairs that match the input criteria. The table includes the number of unique supporting chimeric reads along with the query parameter for reference. For each miRNA–target pair, users can click its “Show detail” link to access more comprehensive information on a detail page. The detail page (Fig. 3) presents the tabular and graphical views of the chimeric reads that support a selected miRNA–target pair and its binding sites. The query setting section first displays the user-defined search settings (Fig. 3a). Then, in the “Interaction Detail (Table View)” section, users can adjust the read depth threshold (default > 0), the binding energy constraint (default < 0), and CIMS presence to further refine the results shown in the detail table (Fig. 3b). Users can modify these filter options as needed. The binding site detail table (Fig. 3c) lists miRNA-binding events across different experiments, showing the binding sites inferred from RNAup and miRanda alongside confidence metrics such as read depths and CIMSs. At the end of the detail page, a transcript atlas (Fig. 3d) provides a visual representation of the miRNA-binding sites on the queried mRNA, which can be downloaded for further analysis.

**Figure 4.**
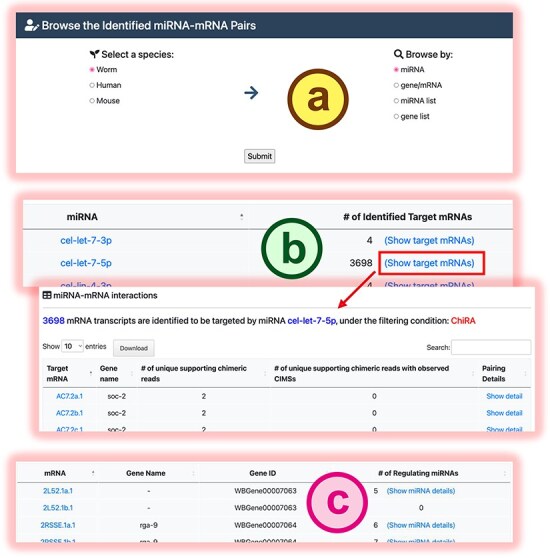
The browse mode in mirTarCLASH. (a) The browse settings. (b) The listed miRNA results when users intend to browse by miRNA. (c) The listed mRNA results when users intend to browse by mRNA. Click the “Show detail” link or the “Show miRNA details” link, and the detailed chimeric read information of the specific miRNA-mRNA pair will be provided as the search detail page.

In the browse mode, users can explore all miRNA targets deposited in mirTarCLASH for a selected species. The browsing options include by miRNAs, by genes, by a specified miRNA list, or by a specified gene list (Fig. 4a). When browsing by miRNA, mirTarCLASH summarizes the number of mRNAs targeted by each miRNA in the CLASH-like experiments (Fig. 4b). Users can click the “Show target mRNAs” link for a specific miRNA to retrieve the list of all mRNAs regulated by this miRNA. Clicking the “Show detail” link for a specific miRNA–target gene pair provides a detailed view of the binding sites, as described in Fig. 3. Alternatively, when browsing by gene/mRNA, mirTarCLASH lists all mRNAs for a selected species and summarizes the numbers of miRNAs that regulate each mRNA (Fig. 4c). Users can click the “Show miRNA details” link for a specific mRNA to access the browse detail page, which allows navigation through the miRNA–target pairs for the selected mRNA. On this detail page, users can also investigate the binding site details in both tabular and transcript graphical views, similar to the detail page provided by the search function (as in Fig. 3).

### A walk-through example and case study

In this section, we demonstrate the biological applicability of mirTarCLASH using a well-known example from *C. elegans*. As a case study and a walk-through example, we examined the binding targets of miRNA *cel-let-7-5p* collected in mirTarCLASH. We first typed in *cel-let-7-5p* in the search mode of mirTarCLASH, and as shown in Fig. 2b, its target genes of *cel-let-7-5p* include several heterochronic genes that are differentially expressed during development. One such target, *lin-41*, was selected for further investigation. In the transcript C12C8.3a.2 of *lin-41*, 597 unique chimeric reads were identified from three independent iCLIP-seq experiments. Among these, 41 reads contain CIMSs, indicating high confidence binding events. We focused on these 41 confident binding sites of *cel-let-7-5p*. Two major binding regions on C12C8.3a.2 were found (Fig. 3c and d): transcript locations 4153–4176 and 4202–4224. These two identified binding site regions coincide with the previously reported *let-7* complementary sites (LCS) 1 and LCS2 [29]. In prior research, *let-7* was shown to target *lin-41* via the binding sites LCS1 and LCS2, controlling the transition from the larvae stage to the adult stage [30]. In summary, this case study indicates that mirTarCLASH can reliably identify miRNA–target genes with confident binding sites, reinforcing its biological relevance in miRNA targeting research.

### Comparison with related works

miRNA regulation is extensively studied, yet most miRNA–target interaction data remain fragmented across the literature. Several databases have been developed to aggregate these existing experimentally validated miRNA–target gene pairs. Among them, DIANA-TarBase [16] and miRTarBase [17] are the most widely used. These two databases deposit a large amount of information about miRNA–target gene pairs for diverse species. However, they do not provide the exact miRNA-binding sites. In contrast, mirTarCLASH is the first database specifically devoted to collecting miRNA–target gene pairs along with the precise binding site regions. We compared mirTarCLASH with DIANA-TarBase and miRTarBase to further highlight the unique features implemented in this research. The comparison is summarized in Table 2.

**Table 2.** The content comparison of mirTarCLASH with existing miRNA–target databases

	Target	High-throughput experiments	No. of chimera analysis methods	No. of RNA–RNA interaction identification method	Confidence analysis of the pairs	Binding site visualization
miRTarBase	Gene-level targets	Only CLIP-seq	By prediction	–	–	–
DIANA-TarBase	Gene-level targets	CLIP-seq and CLASH	1	1	–	–
mirTarCLASH	Gene-level targets and transcript-level targets	Chimeric read-based results	1	2	Read depths, binding energies, number of identifying algorithms, and CIMSs	V

DIANA-TarBase is the first miRNA–target database developed based on human curation. It provides comprehensive details for miRNA–target gene pairs, including the experimental methods, cell types, tissues, regulation effects (positive/negative), and the supporting literature. TarBase enables a complete query interface, allowing users to look up different miRNA–target gene pairs under specific cellular conditions. It also incorporates the analysis results from the microCLIP tool to expand its contents. In its latest version, CLASH, qCLASH, and knockdown experimental results have also been included. Despite these advancements, several limitations remain in the current version of TarBase. In TarBase, the miRNA–target pairs identified from CLIP-seq experiments rely solely on predictions from microCLIP, rather than direct experimental validation. Moreover, for miRNA–target pairs collected from CLASH experiments, no confidence evaluation metrics are available for the identified binding sites. Lastly, TarBase lacks visualization features, making it difficult for users to compare miRNA-binding sites across transcripts. miRTarBase collects miRNA–target gene pairs from two primary sources. The first source is a literature corpus screened by natural language processing techniques. For articles identified to be relevant to miRNA targeting, miRTarBase resorted to human curators to extract the miRNA–target gene pairs studied in these publications. The second source includes CLIP-seq experimental data that probe miRNA-binding events. These CLIP-seq datasets were analyzed by miRTarCLIP [31] in miRTarBase. Since CLIP experiments do not directly reveal the miRNAs responsible for targeting specific mRNAs, the results in miRTarBase consist of only miRTarCLIP-predicted miRNA–mRNA interactions rather than experimentally validated pairs. In addition, the exact binding sites are currently not feasible in miRTarBase. As a result, additional analysis tools and experimental validation are still required to determine the miRNA-binding sites.

In this research, mirTarCLASH was developed through *de novo* analyses of published raw sequencing reads from CLASH-like experiments that probe miRNA-binding events. These CLASH-like experiments were comprehensively analyzed using ChiRA. Unlike prediction-based approaches, mirTarCLASH extracted the miRNA–target segment interactions directly from chimeric reads, eliminating the need for miRNA identity inference. Furthermore, the exact binding sites for each of them are determined in mirTarCLASH using two independent algorithms for cross-referencing, enhancing reliability. In total, mirTarCLASH incorporates the largest collection of CLASH-like experiments across multiple species, including 42/26/5 experiments with 502 061/322 707/224 452 unique hybrid reads for humans/mice/worms (Table 1). It also employs the most comprehensive analysis pipeline, implementing confidence evaluation metrics such as the read depth, the number of identifying algorithms, and the presence of CIMSs. These metrics enable users to select highly confident miRNA–target gene pairs and their corresponding binding sites for further analysis. To improve binding site visualization, mirTarCLASH provides both a tabular view and a transcript graphical view of them, offering an intuitive overview of the miRNA-binding events within a given transcript. In summary, mirTarCLASH delivers comprehensive miRNA–target gene information, complemented by graphical binding site atlases for users to further explore potential miRNA regulation mechanisms.

### Issues with mirTarCLASH

mirTarCLASH is the first miRNA-binding event database primarily based on CLASH-like experiments. Nonetheless, since CLASH-like experiments have only been conducted under limited cellular conditions, the identified miRNA-binding events are restricted to some specific cell types and conditions. Another limitation of mirTarCLASH is that it currently supports only three species (humans, mice, and worms). As more CLASH-like experiments become available for additional species, we plan to expand mirTarCLASH to incorporate a broader range of species. Upcoming public-available CLASH datasets for miRNA targeting investigation under different cell conditions will be collected and analyzed in the future, and mirTarCLASH will be updated accordingly. Finally, for researchers conducting their own CLASH experiments relevant to miRNA targeting, we provide a CLASH data analysis pipeline simplified from our recent work (MUTACLASH [32]). The download link for this simplified tool can be found in the “Data and tool availability” section.

## Conclusions

In this research, we constructed the mirTarCLASH database that collects experimentally probed miRNA–target gene pairs and their corresponding binding sites from chimeric read-based experiments in humans, mice, and worms. Based on one chimera analysis tool and two pairing energy calculation programs, we identified and integrated novel miRNA–target interactions and binding sites into mirTarCLASH. To enhance the confidence of the inferred miRNA-binding sites, we further implemented filters that account for the information on read depths, the number of supporting algorithms, and the presence of CIMSs on the chimeric reads. From a well-characterized miRNA–target gene example, mirTarCLASH is suggested to be biologically applicable in studying miRNA-binding events. With an easy-to-use interface and graphical visualization of the deposited binding sites, mirTarCLASH allows users to explore miRNA targeting mechanisms in detail, contributing to a deeper understanding of cellular miRNA regulation.

## Data Availability

The miRNA–mRNA pairs extracted by ChiRA for humans, mice, and worms can be downloaded at https://cosbi.ee.ncku.edu.tw/MirTarClash/download/. The CLASH data processing pipeline simplified from our current work (MUTACLASH) can be referenced at https://github.com/cosbi-nckuee/MirTarClash.
